# Physical Environment vs. Social Environment: What Factors of Age-Friendliness Predict Subjective Well-Being in Men and Women?

**DOI:** 10.3390/ijerph18020798

**Published:** 2021-01-19

**Authors:** Elena del Barrio, Sandra Pinzón, Sara Marsillas, Francisco Garrido

**Affiliations:** 1 Matia Institute of Gerontology, C/ Orense 6, 28020 Madrid, Spain; sara.marsillas@matiafundazioa.eus; 2 Andalusian School of Public Health, Cuesta del Observatorio, 4, 18011 Granada, Spain; sandra.pinzon.easp@juntadeandalucia.es; 3 Department of Criminal Law, Philosophy of Law, Moral Philosophy and Philosophy, University of Jaen, Carretera Bailen, 12, 23009 Jaén, Spain; fpena@ujaen.es

**Keywords:** age-friendly cities, well-being, older people, participation, physical environment, social environment, gender

## Abstract

“Age-Friendly Cities and Communities” is an initiative launched by the WHO in 2007 that has spread to more than 1000 cities and communities around the world. This initiative is based on an integrated physical and social environment for older people, and a model of participatory, collaborative governance. An enabling social environment setting is just as important as material conditions in determining well-being in later life. The objective of this study is to analyze the interaction between age-friendliness (physical and social) and subjective well-being in women and men aged 55 and over in the Basque Country. The methodology was based on a survey of a representative sample (*n* = 2469 individuals). In order to know the predictive power of age-friendliness over subjective well-being, linear regression models separated by gender were constructed. The predictive models of age-friendliness are composed by different variables for men and women. In both cases, the physical environment variables do not remain in the final model. Among the predictors of well-being in men, the coexistence stands out as a safety and support network. In women, the neighborhood has proved to be a very important resource. The conclusions of this study contribute to literature and interventions promoting more effective strategies that enhance older people well-being, considering the gender perspective.

## 1. Introduction

The development of age-friendly cities and communities, adapted to older people’s needs, has become an important area of work in the fields of health, ageing, and public policy. This is the result of several trends, including the complexity of demographic change, the policy objective of supporting the maintenance of people in their homes for as long as possible [1] and the recognition of the role of the environment in active and healthy ageing [2].

In this sense, the “Age-Friendly Cities and Communities” initiative launched by the WHO in 2007 has been extended to more than a thousand cities and communities worldwide [3], highlighting the importance of the systematic and inclusive approach in generating enabling environments [4]. In the Basque Country, the age-friendliness movement began in 2009 with the adhesion of the capitals to the initiative launched by the WHO. In 2012 the Basque Government launched a project at territorial level (Euskadi Lagunkoia-Age-Friendly Basque Country), which currently includes more than 60 municipalities [5]. Since then, age-friendliness has become one of its political strategies on ageing [6].

An age-friendly community is defined as a place where older people are actively involved, valued, and supported with infrastructure and services that are effectively tailored to their needs [7].

Making cities and communities age-friendly ensures that they are inclusive and equitable places, leaving no one behind, especially the most vulnerable older people [4]. Age-friendliness is primarily characterized by the adaptation of mutually reinforcing social and physical environments, by a participatory model of collaborative governance and, above all, by inclusion [8]. This emphasizes an underlying assumption that is now widely shared by policymakers: An enabling social environment is as important as the material conditions in determining the well-being of older people’s lives [9].

Research has shown that age-friendly environments are associated with higher levels of well-being and quality of life in older people [10,11,12,13]. It has even been concluded that older people who perceive their environments as age-friendly are almost four times more likely to report a better quality of life than those who report lower levels of age-friendliness [11]. Its positive association to well-being is not surprising, as the criteria for an age-friendly environment align almost perfectly with both concepts [13]. However, much existing environmental gerontological research has focused on the indoor settings, mainly homes and overlooked wider contexts such as neighborhoods and communities [14,15]. More recently, some studies have extensively examined the multidimensional aspects of the environment [15,16]. Van Dijk [15] explored the relationship of social characteristics of the neighborhood, such as social capital, social cohesion, and social support with well-being. Park and Lee [14] found that after controlling for demographic covariates, physical and social environment features are significantly related to the life satisfaction of older people in Korea. This finding is an empirical basis for identifying those aspects of the environment that can serve as modifiable resources and improve the psychological well-being of older people, in this case, particularly the most vulnerable ones.

Despite these conclusions, there is still insufficient understanding of the actual holistic effects of interventions on the physical and social environments [9,17]. More evidence needs to be generated about how improvements in both settings affect the health and well-being of older people [8]. One of the main challenges facing all community initiatives–either related to ageing or not-is how to assess their impact on individuals and groups [18]. The term “age-friendly” is used when considering how various aspects of a community promote or reduce the health and well-being of individuals as they age [19]. However, scientific research continues to investigate why some places are more age-friendly than others and how age-friendliness relates to the well-being of older people [8].

Focusing on well-being, there is a growing interest in the variables that influence the subjective well-being of older people. Some of the individual factors related to well-being in old age have been highlighted in previous research. Some of these studies conclude that well-being was strongly related to socio-demographic [20]; socio-economic [21,22]; health [23,24]; lifestyle [25,26]; psychological characteristics [27,28]; as well as social relations [22].

Gender is one of the most relevant socio-demographic characteristic in old age. Growing old is not the same for men and women [29]. Sex and gender are important determinants of health and wellbeing [30]. Gender is the characteristic with enough evidence to suggest that there is different wellbeing or survival relationships for older men and women [31]. To explain social phenomena, the Gender Perspective takes into account the differentiated or egalitarian situation, depending on belonging to one sex or the other [29]. Gender is an explanatory social category, also constructed, that helps to understand what lies behind biological sex. Gender, as a social construction, reveals differences in thinking, roles, health, economics, politics, and labor and in old age, these differences are even bigger. These inequalities do not appear in the last stages of life, but are nourished throughout the life cycle and are therefore continuous [32].

Although the Framework for Action on Active Ageing highlighted gender as a cross-cutting determinant, research on ageing from a gender perspective is still limited [29]. The gap in women’s representation in human studies has been well documented [33]. Gender-based analysis is designed to identify the sources and consequences of inequalities between women and men and to develop strategies to address them [30].

In this context, the general objective of this study is to analyze the interaction between age-friendliness, subjective well-being, and gender, specifically regarding the perception of age-friendliness of the physical and social environment, among men and women aged 55 and over in the Basque Country. Variables of perception of the eight areas of age-friendliness, ranging from public spaces, housing, transport, social participation and employment, civic participation, respect and social inclusion, communication and information, and social and health services, are included.

## 2. Materials and Methods

Although the WHO has made several documents available to facilitate the measurement of age-friendliness [34,35], there is no official quantitative tool yet. One of the challenges in evaluating age-friendly city initiatives is to identify an evidence-based approach [36,37,38]. There is a great need for monitoring, evaluating, measuring and assessing the age-friendliness of cities and communities [38]. Despite these challenges, in recent years different researchers have developed a number of tools to measure and evaluate these initiatives in a quantitative way [38,39,40,41,42,43,44].

In this study, however, the tool for measuring friendliness is based on a survey of the living conditions of people aged 55 and over carried out periodically in the Basque Country. This means it does not count on a tool designed ad hoc, but it includes a series of relevant indicators related to the areas of age-friendliness indicated by the WHO [45].

The methodology of this study was based on a survey of a representative sample of community-dwelling residents aged 55 and over in Basque Country (739,231 people aged 55 and over, 33.8% of the total population). Structured interviews were conducted through face to face survey based on a questionnaire assisted by computer. Sampling selection was made through stratified random sampling considering geographic area and age group (55–64 years-old, 65–69, and 80 and over) as main criteria of stratification. Sample distribution followed a proportional method for territory strata and quotas according to age group (55–64, 65–79, and 80 and over) and gender were applied. Households in each stratum were chosen by random selection of those with one person aged 55 and over, only interviewing one person per household. Sample size was determined by required level of disaggregation. Statistics on ageing generally categorize older people as being above a certain age threshold. Indeed, the United Nations (UN) defined older people as those aged 60 years or more in World Population Ageing 2013, while the WHO states that older people in developed world economies are commonly defined as those aged 65 years or more. The WHO also uses an alternative definition, whereby an older person is defined as someone who has passed the median life expectancy at birth [46]. The age selected for analysis, 55 and over, is one of the most widely used cohorts for the study of the ageing population from research and public policy (for example: The Active Ageing Index). Although, there is still much debate about the redefinition of the threshold for the onset of old age because of its implications for the design of public policies and for the social perception of old age [47].

The sample was composed of 2469 individuals (1177 men and 1319 women). Their ages ranged from 55 to 102 years old, with 69.36 (SD = 9.9) being the average age. Regarding marital status, 61% of the participants were married, 9% were single, 6% were divorced, and 24% were widowed (Table 1).

Anonymity and confidentiality of the answers were guaranteed and participation in the study was voluntary. All subjects gave their informed consent for inclusion before they participated in the study and the protocol was approved by the Ethics Committee of University of Jaen (MAY.13/3.TES).

The questionnaire includes items and scales that explore perceptions in relation to areas of age-friendliness (age-friendliness components). The WHO’s subjective well-being scale (WHO5 Well Being Index -1998) is also included for this analysis.

To find out the predictive power of the components of age-friendliness on the subjective well-being of men and women aged 55 and over in the Basque Country, two multiple linear regression models have been carried out, using the WHO5 Well Being Index as the dependent variable [48], and both the predictors of well-being according to the von Humboldt and Leal [49] categories (16 variables) and the selected components of age-friendliness (34 variables) as independent variables

Subjective well-being is a general term used to describe the level of well-being experience of people according to subjective assessments of their lives [49]. It was measured using the WHO-5 Well Being Index -1998 version, a five-item self-administered scale, Likert-type scale with response options of 0 to 5. This scale exclusively measures the positive aspects of the psychology of well-being in short non-invasive questions. It is one of the most widely used questionnaires to assess subjective psychological well-being [50].

The validity of construct of the WHO-5 was analyzed with the item response theory model formulated by Rasch (2012) in older people [51], which confirmed that the 5 items constitute a one-dimensional scale, where each item adds unique information on the level of well-being [50]. A total score can be obtained by adding up all items, with a range of scores from 0 to 100 covering from a total absence of well-being to the highest conceivable level of well-being [50].

In the field of later life, the WHO-5 scale has been validated in different studies for the detection of depression [52,53,54], apathy [55], or suicide [52]. An internal and external validation of the scale has also been carried out in the general older population [56], concluding that the WHO-5 is a useful instrument for the identification of people with reduced subjective quality of life.

This study analyzed the psychometric nature of the scale used to ensure the quality of the measurement. This was done through the analysis of internal consistency as well as through the performance of exploratory factor analyses of main components with Varimax rotation.

Regarding linear regression analyses, in order to control the predictive values of the age-friendliness components by the factors that has been identified by literature and evidence as predictors of wellbeing [49], some independent variables were included such as age, educational level, habitat, marital status, type of household, or health status, among others. These are drawn from several categories that are divided into: Social support; socio-demographic factors; health status; and psychological factors. Moreover, a total of 34 variables related to the 8 age-friendliness areas described by WHO have been included as age-friendliness components (Table 2). The number of indicators available in the survey related to each domain is different, which means that there are domains with 10 indicators and others where only one indicator has been found. The variables in this study encompassed within the physical environment are those included in the areas of outdoor spaces, housing, transport, communication, and health services (Areas 1, 2, 3, 7, and 8). The variables considered in the social environment are those in the areas of social participation, respect, and social inclusion, and citizen participation and employment (Areas 4, 5 and 6). The indicators corresponding to the age-friendliness components proposed by the WHO were included in the linear regression model to explore their association to wellbeing.

In a first phase, a descriptive analysis of the variables separated by men and women was carried out, following the recommendations of Calvente, Rodrigo, and Morante [57], to observe the gender gaps. For the analysis of the quantitative variables, the basic statistics (mean and standard deviation) were used. For categorical variables, the relative frequency distribution with 95% confidence intervals was used.

In order to know the relationship of subjective well-being with the rest of the variables, an analysis of variance was carried out to determine the predictors of subjective well-being, by gender. In order to know the predictive power of age-friendliness over subjective well-being, linear regression models separated by gender were constructed. Then, variables that did not contribute to explain this relationship were eliminated until the final models were obtained.

## 3. Results

### 3.1. Subjective Well-Being

Subjective wellbeing showed satisfactory psychometric properties, in terms of internal consistency (alfa: 0.87). Regarding construct validity (KMO: 0.85), a single factor has been obtained explaining 67.35% of the variance.

Considering descriptive results, the average well-being score was 64.12, with a standard deviation of 22.24 points (Scale of 0–100). Men have a higher well-being index ($\overset{-}{x}$ = 67.9) than women ($\overset{-}{x}$ = 61.2).

### 3.2. Factors Associated with Well-Being by Gender

The gender-segregated analysis showed that the factors associated with subjective well-being are different for men and women. However, most of these factors have statistically positive results in their relationship to subjective well-being in both genders (*p* < 0.005) (Table 3).

### 3.3. Subjective Well-Being and Age-Friendliness Components by Gender

The gender-segregated analysis showed that the components of age-friendliness are different for men and women (Table 4). For instance, more housing, social participation and communication, and information indicators related to subjective well-being in case of women compared to men. Concerning outdoors spaces, barriers in the immediate environment and difficulty of access to the supermarket or grocery shop are associated with subjective well-being in both women and men. In the latter, the difficulty of access to the park or green area is also associated, although with little significant relevance. In the area of housing, subjective well-being is related with barriers within the home for both men and women. For women, barriers to entry and tenure are also associated with well-being. In the area of social participation, there is a direct association between the performance of all activities and women’s subjective well-being. Among men, no association was found with attendance at religious events or educational activities. In the area of citizen participation and employment, for men, subjective well-being is associated with the practice of activities in the field of social and community services, associations of all kinds and political parties. In the case of women, subjective wellbeing is associated with all kinds of these voluntary practices except participation in parish groups, and also participation in the labor market. In the area of communication, the outstanding gender difference is that compared to men, the case of women having a mobile phone is also associated with their well-being, in addition to having a computer/tablet and access to and use of the Internet, which are significant in both genders.

### 3.4. Predictors of Subjective Well-Being by Gender

In order to identify the predictors of the subjective well-being in men and women, different regression models have been carried out. The same variables, selected by literature review, have been included in the initial models for each gender, but those that have obtained significant results for each have been maintained in the final models.

#### 3.4.1. Predictors of Subjective Well-Being in Men

In order to identify the predictors of the subjective well-being in men, several regression analyses were carried out: Firstly, incorporating in the model the factors associated with well-being that had obtained significant results; secondly, the components of age-friendliness.

In the first of the regressions, 7 out of 12 variables were selected as potentials for the final model (corrected R square = 0.232). The variables selected were habitat (Beta -0.068; *p* = 0.019); type of household (Beta 0.065; *p* = 0.026); satisfaction with personal relationships (Beta 0.088; *p* = 0.031); health status (Beta 0.206; *p* < 0.000); need for help (Beta -0.071; *p* = 0.017); satisfaction with achievements (Beta 0.128; *p* = 0.002); and satisfaction with how safe and secure they feel (Beta 0.176; *p* < 0.000).

Regarding the components of age-friendliness, seven were selected to be introduced in the final model (corrected R square = 0.142). These were perform physical or sport activities (Beta 0.108; *p* < 0.000); perform leisure-housing activities (Beta 0.078; *p* = 0.011); perform social activities (Beta 0.114; *p* < 0.000); perform tourism, travel activities (Beta 0.114; *p* < 0.000); satisfaction with his/her feeling of belonging to the community or group of people (Beta 0.198; *p* = 0.000); participates in educational, cultural, gastronomic associations (Beta 0.071; *p* = 0.021); and having difficulty in accessing the health center (Beta -0.093; *p* = 0.003).

In the regression analysis for the final model, the variables resulting from the previous regressions were included. The final model in men was constructed with nine variables, four related to the components of age-friendliness (corrected R square = 0.247) (Table 5).

Considering collinearity, two variables obtained high results in the indices but below the recommended threshold of 30, so they remained in the analysis [58].

By including age, marital status, educational level, and economic level as control variables in the final model, the variable “lives with others” leaves the model (Sig = 0.083; Beta 0.074); but none of these variables are significant in the final model

#### 3.4.2. Predictors of Subjective Well-Being in Women

In order to identify the predictors of the subjective well-being in women, several regression analyses were carried out: Firstly incorporating in the model the factors associated to the well-being, which obtained significant results; secondly, the components of age-friendliness.

In the first of the regressions, 8 out of 15 variables were selected as potentials for the final model (corrected R square = 0.326). The variables selected were habitat (Beta -0.072; *p* = 0.006); origin (Beta 0.075; *p* = 0.004); social network contact (Beta 0.088; *p* = 0.001); satisfaction with personal relationships (Beta 0.098; *p* = 0.003); health status (Beta 0.242; *p* < 0.000); need for help for ADLs (Beta -0.130; *p* < 0.000); satisfaction with the achievements she is making in life (Beta 0.191; *p* < 0.000); and satisfaction with her security regarding her future (Beta 0.108; *p* = 0.001).

Considering the components of age-friendliness, seven were selected to be introduced in the final model (corrected R square: 0.234): Barriers in public transport (Beta-0.112; *p* < 0.000); performance of physical or sports activities (Beta 0.105; *p* < 0.000;); social activities (Beta 0.076; *p* = 0. 011); doing tourism and/or travel (Beta 0.142; *p* < 0.000); satisfaction with their feeling of belonging to a community or group of people (Beta 0.294; *p* < 0.000); participation in neighborhood associations (Beta 0.083; *p* = 0.002); and labor market participation (Beta 0.089; *p* = 0.001).

In the regression analysis for the final model, the variables resulting from the previous regressions were included. The final model in women was constructed with 11 variables, 4 of them related to the components of age-friendliness (corrected R2: 0.323) (Table 6).

As in the male model, two variables obtained high results in collinearity indices but below the recommended threshold of 30, so they remain in the analysis [58].

By including age, marital status, educational level, and economic level as control variables in the final model, the variable “born in the Basque Country” leaves the model (Sig = 0.051; Beta 0.052); but none of these variables are significant in the final model.

## 4. Discussion

Gender is a characteristic with enough evidence, suggesting that there is different well-being or survival relationships for men and women [31]. For instance, Lennartsson and Silverstein [59] found that solitary activities reduced mortality risk for men but not for women, while Warr, Butcher, and Robertson [60] found that family and social relationships influenced more women’s well-being compared to men. Agahi and Parker in Adams and colleagues [31] also found that women’s mortality risk decreased with social activities, whereas men’s risk was higher with solitary hobbies and gardening, for example. This previous evidence highlights the importance of analyzing subjective well-being in men and women separately in order to clarify what influences and predicts the well-being of both.

The final model obtained in men is composed of nine predictors, four of which are included in the components of age-friendliness, particularly social and citizen participation. The other variables included are relevant factors selected from scientific evidence where social support, health status, and psychological factors predominate. Thus, the variables predicting men’s well-being are living with other people, being satisfied with personal relationships, being in good health, being satisfied with their achievements and with how safe and protected they feel, as well as carrying out physical exercise, domestic and social leisure activities, and participating in associations of a gastronomic, educational, cultural, or sporting nature.

Regarding women, the final model consists of 11 factors, 4 belonging to the components of age-friendliness within the areas of social participation, citizen participation, and social inclusion. The other includes socio-demographic, social support, health, and psychological variables. Living in a rural environment (less than 20,000 inhabitants); having been born in the Basque Country; maintaining contact with family, friends and/or neighbor; being satisfied with their personal relationships; being in good health; not needing help for the DLA; being satisfied with their achievements; doing physical exercise; tourism or travel; being satisfied with their feeling of belonging to the community; and participating in neighborhood associations, predicts higher levels of subjective well-being in women.

The results, therefore, have shown that only for women socio-demographic factors such as habitat or origin are relevant, leaving out other important variables such as age, marital status, wealth, or educational level. These findings contradict previous studies where some demographic variables, such as age, income, work status, marital status, and educational level [61] are associated with subjective well-being. However, these studies have also revealed that these variables only explain a partial amount of the variance in well-being [61]. This means that the weight of other variables is very important and could explain, to a greater extent, individual differences in levels of well-being and the subjective nature of the concept [61]. Additionally, subjective well-being as an overall measure does not seem to undergo significant changes associated with age, either in cross-sectional or in longitudinal studies [62]. On the other hand, educational level does not seem to be relevant either when controlling for other factors, and it is possible that education may exert indirect relations to subjective well-being through a mediating role [61]. With regard to the level of wealth or income, it has also been concluded that countries with the highest level of wealth have almost no correlation between this variable and well-being [61,63,64,65,66].

Sociodemographic variables have been maintained as predictors of subjective well-being only in women, being origin (place of birth) and rurality (less than 20,000 inhabitants). Regarding rurality, some researchers have analyzed the differences between urban and rural communities related to well-being, but scarce evidence is found in terms of ageing population [67]. According to Van Hoof and Kazak [67], some studies have corroborated that rural areas approach or exceed urban areas in terms of life satisfaction or well-being. However, the higher the density of urban settlement, the greater the proximity to public services, influencing the quality of life of people and being especially relevant for older people [67]. In the literature, findings about environment and health focus largely on urban areas, however, how the age-friendly community characteristics are related to rural environments is less known [39]. Research of well-being in rural women has also been relatively scarce [68]; for instance, some authors found a relationship between habitat and life satisfaction in women, in which rural women obtained higher life satisfaction than urban women, although not significantly [68]. In this study, women who live in areas with fewer than 20,000 inhabitants were more likely to report higher levels of well-being. A possible explanation may be related to the conclusions of previous research in which satisfaction with social relations is higher among older people living in rural environments [69], due to in less populated areas people know each other better. Neighbors are a very important type of relationships in villages, especially for women [62]. Studies such as Bosch and Gómez [70] show that women have a deeply rooted role and caring function, and in rural environments they find support to continue to carry out their role in “good neighbor” networks in an adequate and satisfactory way.

Origin, a socio-demographic predictor of women’s well-being, refers to having been born in the Basque Country. This variable could be understood in relation to the importance of the community and belonging to the place, another predictor variable of well-being in women. As women age, their dependence on their neighborhood increases [71] and their residential stability is associated with attachment to the community [71,72].

Regarding men’s well-being, socio-demographic variables have not remained as predictors. However, social support factors emerged as predictors, for instance, living with other people. For men, living in a household with other people, i.e., living together, has a positive effect on their well-being. This type of social support is very important because of their relative poorer performance and independence in leading an autonomous life on their own, at least compared to women [73]. The assignment of traditional gender roles leads to a men’s higher level of dependence in domestic tasks, which implies the need to live with other people in order to perform them. Several studies show how living together as a couple entail higher life satisfaction, better emotions, physical and mental health, economic resources, and social integration, support, and relations [74]. Additionally, marriage is one of the strongest predictors of subjective well-being: Married people report a higher degree of life satisfaction than single, widowed, or divorced ones [61,65,66,67].

Another predictor of well-being, in this case for men and women, is satisfaction with personal relationships. Good relationships with family, friends, and other people in their social network provide higher indices of subjective well-being in both genders. Social support networks have been identified as important influences on the affective and cognitive components of well-being [75]. Furthermore, protective effects of social networks on morbidity and mortality have been strongly recognized [71,76]. This correlation between various types of social support and well-being in older people [19] is considered a consistent result.

Health predominates among other predictors of subjective well-being. The relationship between health and well-being has also been found in multiple studies [19,22,77,78]. Menec and Nowicki [39] found that perceived health had significant positive effects on the physical environment, social environment, opportunities for participation, and transport options. In our study, perceived health is the strongest predictor in the final models for men and women.

In addition, in women, the situation of dependence or autonomy remains together with health. For them, not needing help to carry out the daily life activities is also a clear predictor of higher subjective well-being. Functional limitations of older people have been found to be associated with it [61,78] and some studies have corroborated how this affects women more [61]. These results may be related to the importance of living together for men, but not for women. In this sense, older women, more “autonomous” in terms of not needing other people to run their home, can find greater obstacles in functional dependence situations, decreasing their subjective well-being.

On the other hand, in men, satisfaction with feelings of safety and protection is included as one of the psychological factors predicting well-being. These results can be analyzed in coherence with the previous ones on coexistence. For their greater well-being, men need to perceive a safety network that begins with live as a couple. In later life “the family becomes the great substitute for employment as a source of sociability, identity and self-esteem or time structuring” ([79], p. 115). When men retire, they move from a socially open relationship in a working environment, which provides them with references of identity, prestige, friendships, solidarity and so on, to a state that requires them to adjust to new developments in the world of marriage and the family [80]. In women, however, the continuity of the role can have positive consequences, with a better psycho-social adjustment at this life stage.

Moreover, satisfaction with the achievements made in life is one of the psychological factors emerged as predictor of well-being for both men and women. In this sense, self-esteem and self-efficacy have positive relationships with the highest level of well-being [61]. Regarding self-efficacy, Gómez et al. [81] indicate that the more confident a person feels in achieving their goals and objectives in life, the higher level of subjective well-being experiences. According to Diener et al. [65], the relationship between subjective well-being and goals is mediated not only by the fact that people have clear goals, but also by the progress made in achieving them [62]. At the individual level, the continuity or replacement of roles through participation in appropriate activities and with family support can contribute to the individual’s sense of meaning or purpose and the maintenance of a sense of identity [31]. Participation in activities involves the pursuit or achievement of personal goals, thus adding a sense of personal mastery or achievement [31,82].

Therefore, the activities carried out by people take on special relevance for the perception of well-being. The other large group of variables included in the model presented in this study are those age-friendliness components described above. Of the eight areas of age-friendliness, only few remain in the model for predicting well-being in men and women, mainly social and citizen participation.

Among the social participation activities predicting well-being for men and women, physical or sporting activities such as doing sport or exercise, walking, going to the mountains, etc., predominate. Several studies have already confirmed this positive relationship between the practice of physical exercise and the feeling of well-being and personal satisfaction [61,83,84], and even effects have been found on physical and psychological health in older people [85].

In men, it has also been identified that participation in domestic leisure activities or hobbies, such as gardening, handicrafts, DIY, etc., is a predictor of well-being. These activities, commonly more solitary, have obtained contradictory results in previous research [86], although a recent study has found that “solitary-active” activity is associated with a reduction in mortality risk in men [59].

Other types of activities that favor the subjective well-being of men are those of a social nature such as going to the bar, meeting friends, going outdoors to have lunch or dinner, etc. These type of social participation and social contacts have positive correlations with personal well-being measures, as confirmed by previous research [86] and from which the ageing theory of activity emerges [87]. The review conducted by Adams et al. [31] confirms that informal social participation, such as visiting friends, has a positive outcome in relation to well-being in old age. According to these authors, strong evidence relating social activity with positive well-being, as inherent social intimacy seems, to be a very important, if not the most important, aspect of engagement that influences people’s well-being [31].

However, for women, tourism, travel, and/or excursions are the social participation activities that predict subjective well-being. These results are corroborated by previous studies that have shown how travelling in old age affects physical, psychological, social, and spiritual dimensions such as integration, acceptance, contribution, updating, and coherence; and that this leisure activity offers opportunities for significant participation in adulthood [88,89]. The review done by Morgan et al. [90] finds that tourism can improve the well-being of older people and promote a renewed sense of purpose, facilitating their transition from work to retirement. Several authors have found that tourism has a positive psychological impact on older people, on their well-being, quality of life, perceived health, and life satisfaction, regardless of the type or length of travel [90]. However, few studies analyzed tourism in old age from a gender perspective [88,89]. Liechty et al. [88] found that group travel with other older women promoted a sense of belonging, empowerment, feelings of self-determination, personal growth, positive emotions and has well-being implications.

On the other hand, in the area of age-friendliness that encompasses social inclusion, the variable feeling of belonging was included. This predicts well-being in women, but not in men. Similar results were found in the study of Tiraphat et al. [11], including social trust as one of the predictors of age-friendly environments [11]. Recent studies have shown the positive effects of social capital, as measured by group participation, sense of belonging, and relationship with neighbors [91] on the perceived functional and psychological health of older people. Well-being is also closely related to a sense of community, and this acts as a predictor of social and psychological well-being [92]. With regard to gender, Phillipson et al. [93] found that women were more concerned about the deterioration of social capital, as they fulfil the role of “neighborhood keepers”. So, as women get older, their dependence on their neighborhood increases [71]. The feeling of belonging seems to be related, in turn, to other predictors of well-being in women such as origin and rurality. In Young et al.’s study [76] another variable associated with a greater sense of belonging in women was living alone, that is, those women who lived alone had a greater sense of belonging to the community arguing that they may have developed social support networks to compensate for living alone.

Finally, for both men and women, the resulting regression models had variables from the civic participation component, although the specific indicator differs. For men, it is participation in educational, cultural, sporting, professional, gastronomic, choral, or literary associations, which predict well-being. Conversely, for women the predictor is participation in neighborhood associations. Several studies have found that participation of older people in socially productive activities is associated with well-being [94] as well as meet service needs in the community [15].

The differences between men and women in the type of civic participation are linked to the other predictor variables and the gender differences found throughout this study. Associations of a cultural, sporting, professional, choral, or gastronomic nature are traditionally made up of men in the Basque Country, something that is reflected today in their greater participation in this type of associations [95,96]. Neighborhood associations played a fundamental role in the recovery of areas of sociability in the Basque Country, not only in the field of self-management, the assembly movement, or the control of municipal management, but also in others such as the revitalization or recovery of popular festivals [95]. This type of associative participation being a predictor of women’s participation is a result that is in line with the previous ones. The neighborhood is a space conquered beyond the home, which means many benefits for women, and which is related to other variables such as rurality, origin, or sense of belonging.

Therefore, none of the variables introduced as physical environment areas are kept as predictor variables in the resulting models, in contradiction to other studies that have found that the areas of “outdoor spaces and buildings”, “transport”, “housing”, along with “community support and health services” [15], and “security” [97] seemed to be essential elements for older people and an important goal to prevent them from losing their social and physical well-being [97].

Despite the findings, our study also has some inherent limitations. First, it is based on the use of a survey not specifically designed for the purpose of measuring perceptions of friendliness. For this reason, some indicators may not fully reach to catch the multidimensionality of friendliness components. Even so, this survey has a wide variety of indicators related to this concept, so this limitation can become an opportunity by optimizing the use of this tool for different purposes and to test its potential in the research of this or other fields. Additionally, it is a cross-sectional study, which prevents capturing the dynamics of friendliness and well-being and extracting causal inferences. Therefore, it is not possible to determine their direction of the association based on the findings of this study. However, the results establish a significant association, which is an important step that encourages further studies to identify directionality. On the other hand, although the survey contains a variable to measure the habitat in which the respondent resides, which would allow a multilevel analysis to be made by looking at the differences between the more rural and more urban environments of men and women, the sampling conducted does not provide a sufficient number of people who meet these conditions for a detailed analysis of habitat of less than 20,000 inhabitants. Future research could indeed be developed in order to identify area level effects regarding friendliness.

## 5. Conclusions

Among the predictors of well-being in men, coexistence stands out as a safety and support network. In women, however, the neighborhood has been found to be a very important resource. Both for men and women, good health, satisfaction with personal relationships, achievements, and physical exercise are predictors of wellbeing.

Regarding age-friendliness components and areas, those related to the social environment (social participation, citizen participation, and social inclusion) are the fundamental elements in people’s well-being. Conversely other aspects such as physical environment (outdoor spaces, housing, and transport) and municipal services (communication and information, and health services) seem to be less important. However, fewer variables on social and health services were included in this study compared to social environment ones, which may have influenced the results about their impact on people’s well-being.

Finally, it is important to analyze separately the perceptions of men and women in order to advance in the knowledge of the different realities. This study has contributed to literature by providing regression models including predictors of well-being and age-friendly components separated by gender. In this way, action programs and policies can be designed more concretely, including the gender perspective, in order to promote more effective strategies that enhance their well-being.

## Figures and Tables

**Table 1:** Demographics of participants (*n* = 2469).

	*n*	%
Gender		
Male	1115	45%
Female	1381	55%
Age		
Mean (SD)		69.4 (9.92)
55–64	974	39%
65–79	1044	42%
80+	478	19%
Educational level		
Less than primary education	847	34%
Complete primary studies	799	32%
Secondary and higher education	844	34%
Origins		
Basque Country	1477	59%
Others	1019	41%
Type of dwelling		
Owner-occupant	2314	93%
Private rent	145	6%
Others	34	1%
Marital status		
Single	231	9%
Married or living with a partner	1524	61%
Widowed	601	24%
Separated/Divorced	140	6%
Living together with a partner	1926	37%
Needs help DLAs	503	20%

**Table 2:** Variables selected as components of age-friendliness.

**Area 1. Outdoor Spaces and Buildings**
1. Barriers in the immediate environment (yes/no)
2. Perception of crime, violence or vandalism (yes/no)
3. Difficulty of access to parks and green areas (yes/no)
4. Difficulty of access to supermarket or food shop (yes/no)
**Area 2. Housing**
5. Barriers inside the home (yes/no)
6. Access barriers to the building (yes/no)
7. Tenancy regime (property/other situation)
8. Adapted housing (yes/no)
**Area 3. Transport**
9. Difficulty in accessing public transport (bus, train, etc.) (yes/no)
10. Public transport barriers (bus, train, etc.) (yes/no)
**Area 4. Social participation**
Carrying out of activities: (performed/not performed)
11. Physical or sporting,
12. Domestic leisure,
13. Cultural,
14. Social,
15. Tourism,
16. Religious acts and
17. Educational activities
**Area 5. Respect and social inclusion**
18. Sense of belonging to a community or group of people (0–10)
**Area 6. Civic participation and employment**
Participation in voluntary activities:
19. Social and community services (participation/not participation)
20. Educational, cultural, sports or professional, gastronomic, choral and literary associations (participation/no participation)
21. Social or charitable movements (participation/no participation)
22. Neighborhood associations (participation/no participation)
23. Parish groups (participation/no participation)
24. Other organizations (participation/no participation)
Political participation:
25. Union, political party or political action group meeting (participation/no participation)
26. Attendance at protest or demonstration (participation/no participation)
27. Contact with a politician or public official (participation/no participation)
Employment
28. Relationship with work activity (working, not working).
**Area 7. Communication and information**
29. Availability of mobile phone (yes/no)
30. Availability of land line (yes/no)
31. Computer/Tablet availability (yes/no)
32. Internet access at home (yes/no)
33. Internet use (yes/no)
**Area 8. Health services**
34. Difficulty in accessing the health center (yes/no)

**Table 3:** Subjective well-being according to determinants by gender.

Variable	Male				Female			
	*n*	Mean	σ	*p*	*n*	Mean	σ	*p*
Total	916	68.06	19.99		1170	61.45	22.98	
Age								
55–64	406	69.26	19.7	0.003	452	65.09	22.2	0.000
65–79	418	68.06	20.0		519	61.05	22.7	
80+	109	61.92	22.7		215	53.23	24.8	
Educational Level								
Less than primary education	250	66.28	20.0	0.151	426	56.16	23.4	0.000
Primary education or higher	682	64.43	20.4		759	64.02	22.7	
Wealth level								
Low	34	61.32	25.99	0.000	47	45.00	27.90	0.000
Medium	795	67.60	19.71		1040	61.70	22.33	
High	59	77.98	15.33		62	73.67	20.74	
Habitat								
<20,000 inhabitants	312	70.00	19.2	0.022	366	66.46	21.9	0.000
20,000 inhabitants or more	621	66.79	20.8		820	58.81	23.5	
Origin								
Born outside the Basque Country	372	66.29	21.2	0.053	506	59.10	24.5	0.000
Born in the Basque Country	561	68.91	19.6		680	64.95	21.6	
Married or living together								
Yes	686	69.18	18.9	0.001	644	62.95	22.7	0.004
No	247	64.22	23.4		543	59.06	23.7	
Type of household								
Individual	158	61.94	24.6	0.000	309	58.28	24.5	0.011
Live with other people	775	69.07	19.1		878	62.19	22.7	
Non-presential contact								
No	58	64.71	22.3	0.227	71	49.23	24.9	0.000
Yes	870	68.03	20.2		1110	61.97	23.0	
Satisfaction of personal relationships								
Low	8	29.73	27.95	0.000	15	42.07	23.50	0.000
Medium	398	63.69	20.60		500	53.67	22.67	
Hight	510	72.10	17.90		655	67.83	21.09	
State of health								
Regular, bad or very bad	350	58.94	22.2	0.000	536	50.01	23.3	0.000
Good or very good	583	73.22	16.9		650	70.41	18.7	
Need for assistance DLAs								
No	846	69.07	19.6	0.000	953	65.00	21.5	0.000
Yes	87	56.23	23.1		233	45.58	23.5	
Satisfaction Achieved								
Low	12	32.56	26.13	0.000	27	31.26	19.56	0.000
Medium	501	64.74	20.12		675	56.44	22.38	
High	388	73.55	17.14		444	70.98	19.32	
Safe and secure satisfaction that you feel								
Low	16	40.82	29.74	0.000	13	49.68	26.39	0.000
Medium	455	63.87	20.01		591	55.53	23.18	
High	440	73.78	16.84		558	68.36	20.47	
Satisfaction and confidence in your future								
Low	38	53.75	26.74	0.000	64	46.11	27.20	0.000
Medium	510	66.24	19.5		630	57.29	22.84	
High	323	73.78	16.65		392	71.09	18.05	
Concern about old age								
No	549	69.19	18.9	0.013	524	65.35	21.8	0.000
Yes	367	65.81	21.9		638	57.80	23.9	

**Table 4:** Subjective well-being according to components of age-friendliness by gender.

	Males				Females			
	*n*	Mean	σ	*p*	*n*	Mean	σ	*p*
Outdoor Spaces								
Barriers in the immediate environment	94	63.10	22.5	0.015	180	51.59	27.8	0.000
Perception of unsafe environment	86	68.67	22.7	0.839	147	60.50	25.6	0.649
Difficulty of access to the park or green area	48	63.03	21.4	0.040	72	61.95	23.0	0.259
Difficulty in accessing a supermarket or grocery shop	46	55.7	24.2	0.000	95	48.73	25.8	0.000
Housing								
Home ownership	848	68.27	19.78	0.051	1120	61.51	23.10	0.024
Barriers inside the home	52	61.66	23.6	0.021	101	48.48	25.2	0.000
Barriers in the access to the building	93	64.34	21.5	0.072	186	53.12	27.6	0.000
Adapted housing	416	67.16	19.1	0.354	467	61.85	22.8	0.282
Transport								
Barriers in public transport	55	52.96	23.5	0.000	99	50.34	26.7	0.000
Difficulty in accessing public transport	67	59.91	23.1	0.001	134	45.65	27.6	0.000
Social participation								
Physical activity	875	68.92	25.1	0.000	1028	63.46	21.8	0.000
Domestic leisure activities	404	70.59	19.3	0.000	668	63.72	21.7	0.000
Cultural activities	443	71.68	17.4	0.000	613	67.24	19.8	0.000
Social activities	817	69.69	18.9	0.000	979	64.36	21.6	0.000
Tourism	640	71.14	18.1	0.000	714	67.03	21	0.000
Religious events	445	69.17	20.3	0.060	767	63.31	22.2	0.000
Educational activities	114	68.88	20.1	0.571	175	67.36	20.8	0.000
Respect and inclusion								
Sense of belonging to a community or group								
Low	11	44.86	29.3		29	35.52	26.8	
Medium	461	64.83	21		571	55.81	22.6	
Hight	426	72.65	17.1	0.000	535	69.11	19.9	0.000
Citizen participation and employment								
Social and community services	50	73.84	19.66	0.032	68	69.82	22.99	0.002
ducational, cultural, and gastronomic associations	94	75.78	16.85	0.000	64	70.13	2084	0.001
Social or charitable movement	48	70.46	20.14	0.366	46	70.51	22.31	0.005
Neighborhood associations	47	74.87	16.53	0.015	58	72.00	20.68	0.000
Parish groups	32	66.95	21.52	0.795	65	63.11	24.89	0.492
Other Organizations	29	67.10	23.24	0.836	39	69.00	24.90	0.033
Participation in trade unions	65	73.90	19.20	0.012	38	63.10	25.80	0.591
Participation in events	116	72.10	20.70	0.015	90	65.41	20.50	0.074
Contact with a politician	47	73.41	21.20	0.052	41	60.79	27.90	0.927
Relationship with work activity: Working	167	70.32	18.99	0.084	167	69.22	19.80	0.000
Communication and information								
Mobile phone	834	68.30	20.0	0.059	1004	62.31	22.6	0.000
Land line	830	68.27	19.7	0.088	1095	61.11	23.0	0.738
Computer or tablet	500	70.75	19.0	0.000	562	63.47	22.6	0.002
Internet access	506	70.55	18.8	0.000	565	63.00	22.2	0.013
Internet use	514	64.93	21.3	0.000	776	57.96	23.7	0.000
Health Services								
Difficult access to the health center	46	54.60	24.0	0.000	87	45.80	28.2	0.000

**Table 5:** Model weights of each predictor and their significance on the dependent variable for men.

	Beta	Sig.	95% CI	
Living with others	0.074	0.083	−0.508	8.249
Satisfaction with personal relationships	0.095	0.021	0.199	2.457
Good or very good health	0.211	0.000	6.048	11.087
Satisfaction with achievements in life	0.103	0.013	0.286	2.439
Satisfaction with how safe and secure his feel	0.162	0.000	1.034	3.274
Perform physical or sports activities	0.100	0.001	3.552	13.228
Perform leisure-housing activities	0.066	0.024	0.339	4.908
Perform social activities	0.062	0.039	0.191	7.713
Participate in gastronomic, educational, cultural or sports associations	0.063	0.032	0.337	7.643
Control variables				
Age (65+)	−0,.007	0.809	−2.611	2.037
Wealth level	−0.042	0.166	−4.506	0.776
Educational level (Primary and higher)	0.027	0.409	−0.552	1.353
Marital status(married or living together)	−0.011	0.793	−4.222	3.228

**Table 6:** Model weights of each predictor and their significance on the dependent variable for women.

	Beta	Sig.	95% CI	
Municipality of more than 20,001 inhabitants (Habitat)	−0.075	0.002	−6.547	−1.549
Born in the Basque Country (Origin)	0.060	0.051	−0.006	4.691
Social network contact	0.067	0.005	2.068	11.828
Satisfaction with personal relationships	0.088	0.011	0.330	2.495
Good or very good health	0.237	0.000	7.835	13.095
Need for help for ADLs	−0.089	0.000	−9.768	−2.819
Satisfaction with the achievements in life	0.158	0.000	1.076	2.987
Perform physical or sport activities	0.091	0.001	2.696	9.945
Perform tourism, travel activities	0.101	0.000	2.091	7.194
Satisfaction with her sense of belonging to a community or group of people	0.089	0.020	0.173	1.998
Participation in neighborhood associations	0.065	0.017	1.128	11.684
Control variables				
Age (65+)	−0.022	0.417	−3.420	1.417
Wealth level	0.021	0.444	−1.540	3.514
Educational level (Primary and higher)	0.043	0.119	−0.199	1.741
Marital status(married or living together)	−0.025	0.346	−3.449	1.211

## Data Availability

Restrictions apply to the availability of these data. Data was obtained from Basque Country Government and are available from the authors with the permission of Basque Country Government.

## References

[1] Phillipson C., Buffel T. (2017). Can global cities be age-friendly cities? Urban development and ageing populations. Innov. Aging.

[2] Noordzij J.M., Beenackers M.A., Roux A.V.D., Van Lenthe F.J. (2019). Age-friendly cities: Challenges for future research. Bull. World Health Organ..

[3] World Health Organization (2020). Age-Friendly World. https://extranet.who.int/agefriendlyworld/.

[4] World Health Organization (2018). The Global Network for Age-Friendly Cities and Communities: Looking Back over the Last Decade, Looking Forward to the Next.

[5] Del Barrio E. (2014). Guía de Implantación y Uso en Municipios: Euskadi Lagunkoia.

[6] Gobierno Vasco, Matia Instituto (2015). Estrategia Vasca de Envejecimiento Activo 2015–2020.

[7] Fitzgerald K.G., Caro F.G. (2014). An Overview of Age-Friendly Cities and Communities Around the World. J. Aging Soc. Policy.

[8] Lui C., Everingham J.-A., Warburton J., Cuthill M., Bartlett H. (2009). What makes a community age-friendly: A review of international literature. Australas. J. Ageing.

[9] Buffel T., Phillipson C., Scharf T. (2012). Ageing in urban environments: Developing ‘age-friendly’cities. Crit. Soc. Policy.

[10] Toma A., Hamer M., Shankar A. (2015). Associations between neighborhood perceptions and mental well-being among older adults. Health Place.

[11] Tiraphat S., Peltzer K., Thamma-Aphiphol K., Suthisukon K. (2017). The Role of Age-Friendly Environments on Quality of Life among Thai Older Adults. Int. J. Environ. Res. Public Health.

[12] Nieboer A.P., Cramm J.M. (2017). Age-Friendly Communities Matter for Older People’s Well-Being. J. Happiness Stud..

[13] Gibney S., Zhang M., Brennan C. (2020). Age-friendly environments and psychosocial wellbeing: A study of older urban residents in Ireland. Aging Ment. Health.

[14] Park S., Lee S. (2016). Age-friendly environments and life satisfaction among South Korean elders: Person–environment fit perspective. Aging Ment. Health.

[15] van Dijk H. (2015). Neighbourhoods for Ageing in Place. PhD Thesis.

[16] Wahl H.-W., Schilling O., Oswald F., Iwarsson S. (2009). The home environment and quality of life-related outcomes in advanced old age: Findings of the ENABLE-AGE project. Eur. J. Ageing.

[17] Scharlach A.E., Lehning A.J. (2012). Ageing-friendly communities and social inclusion in the United States of America. Ageing Soc..

[18] Greenfield E.A., Oberlink M.M., Scharlach A.E., Neal M.B., Stafford P.B. (2015). Age-Friendly Community Initiatives: Conceptual Issues and Key Questions. Gerontologist.

[19] Greenfield E.A. (2018). Age-Friendly Initiatives, Social Inequalities, and Spatial Justice. Hast. Cent. Rep..

[20] George L.K. (2009). Still Happy After All These Years: Research Frontiers on Subjective Well-being in Later Life. J. Gerontol. Ser. B.

[21] Larson R. (1978). Thirty years of research on the subjective well-being of older Americans. J. Gerontol..

[22] Pinquart M., Sörensen S. (2000). Influences of socioeconomic status, social network, and competence on subjective well-being in later life: A meta-analysis. Psychol. Aging.

[23] Jones T.G., Rapport L.J., Hanks R.A., Lichtenberg P.A., Telmet K. (2003). Cognitive and psychological predictors of subjective well-being in urban older adults. Clin. Neuropsychol..

[24] Leung B.W.-C., Moneta G.B., McBride-Chang C. (2005). Think Positively and Feel Positively: Optimism and Life Satisfaction in Late Life. Int. J. Aging Hum. Dev..

[25] Sasidharan V., Payne L., Orsega-Smith E., Godbey G. (2006). Older adults’ physical activity participation and perceptions of well-being: Examining the role of social support for leisure. Manag. Leis..

[26] Şener A., Terzioğlu R.G., Karabulut E. (2007). Life satisfaction and leisure activities during men’s retirement: A Turkish sample. Aging Ment. Health.

[27] Ferguson S., Sue J., Goodwin A.D. (2010). Optimism and Well-Being in Older Adults: The Mediating Role of Social Support and Perceived Control. Int. J. Aging Hum. Dev..

[28] Herero V.G., Extremera N. (2010). Daily life activities as mediators of the relationship between personality variables and subjective well-being among older adults. Pers. Individ. Differ..

[29] Fernández-Mayoralas G., Schettini R., Sánchez-Román M., Rojo-Pérez F., Agulló M.S., João Forjaz M. (2018). El Papel Del género En El Buen Envejecer. Una revisión sistemática Desde La Perspectiva científica. Prisma Social.

[30] Heidari S., Babor T.F., De Castro P., Tort S., Curno M. (2019). Equidad según sexo y de género en la investigación: Justificación de las guías SAGER y recomendaciones para su uso. Gac. Sanit..

[31] Adams K.B., Leibbrandt S., Moon H. (2010). A critical review of the literature on social and leisure activity and wellbeing in later life. Ageing Soc..

[32] García R.I.C., Fernández C.C., Cortés C.C. (2012). Envejecer activamente desde una perspectiva de género. Identidades Culturales y Educación en la Sociedad Mundial [Recurso Electrónico].

[33] Coen S., Banister E. (2012). What a Difference Sex and Gender Make: A Gender, Sex and Health Research Casebook.

[34] World Health Organization (2007). The Checklist of Essential Features of Age-Friendly Cities.

[35] World Health Organization (2015). Measuring the Age-Friendliness of Cities: A Guide to Using Core Indicators.

[36] Buckner S., Mattocks C., Rimmer M., LaFortune L. (2018). An evaluation tool for Age-Friendly and Dementia Friendly Communities. Work. Older People.

[37] Buckner S., Pope D., Mattocks C., LaFortune L., Dherani M., Bruce N. (2019). Developing Age-Friendly Cities: An Evidence-Based Evaluation Tool. J. Popul. Ageing.

[38] Dikken J., Hoven R.F.V.D., Van Staalduinen W., Hulsebosch-Janssen L.M., Van Hoof J. (2020). How Older People Experience the Age-Friendliness of Their City: Development of the Age-Friendly Cities and Communities Questionnaire. Int. J. Environ. Res. Public Health.

[39] Menec V., Nowicki S. (2014). Examining the relationship between communities’ ’age-friendliness’ and life satisfaction and self-perceived health in rural Manitoba, Canada. Rural. Remote Health.

[40] Handler S. (2014). A Research & Evaluation Framework for Age-Friendly Cities.

[41] Neal M.B., Wernher I. Evaluating Your Age-Friendly Community Program: A Step-by-Step Guide. https://pdxscholar.library.pdx.edu/cgi/viewcontent.cgi?article=1012&context=aging_pub.

[42] Orpana H., Chawla M., Gallagher E., Escaravage E. (2016). Developing indicators for evaluation of age-friendly communities in Canada: Process and results. Health Promot. Chronic Dis. Prev. Can..

[43] Pinheiro F.A., Diogo M.T., Góis J.E.S., Paúl C. (2015). Age-Friendly Cities Performance Assessment Indicators System Validation. Psychology.

[44] Zaman A.U., Thornton K. (2018). Prioritization of Local Indicators for the Development of an Age-Friendly City: A Community Perspective. Urban Sci..

[45] World Health Organization (2007). Global Age-Friendly Cities: A Guide.

[46] Europe A. (2019). Looking at the Lives of Older People in the EU. https://ec.europa.eu/eurostat/statistics-explained/index.php/Ageing_Europe_-_looking_at_the_lives_of_older_people_in_the_EU.

[47] Abellán García A., Ayala García A., Pujol Rodríguez R. (2017). A Vueltas con el Umbral de Inicio de la Vejez. http://envejecimientoenred.es/vueltas-umbral-inicio-la-vejez/.

[48] WHO (2004). Promoting Mental Health: Concepts, Emerging Evidence, Practice: Summary Report.

[49] von Humboldt S., Leal I. (2014). Qué influye en el bienestar subjetivo de los adultos mayores? Una revision sistematica de la literatura. Rev. Argent. Clín. Psicol..

[50] Topp C.W., Østergaard S.D., Søndergaard S., Bech P. (2015). The WHO-5 Well-Being Index: A Systematic Review of the Literature. Psychother. Psychosom..

[51] Lucas-Carrasco R., Allerup P., Bech P. (2012). The Validity of the WHO-5 as an Early Screening for Apathy in an Elderly Population. Curr. Gerontol. Geriatr. Res..

[52] Heun R., Bonsignore M., Barkow K., Jessen F. (2001). Validity of the five-item WHO Well-Being Index (WHO-5) in an elderly population. Eur. Arch. Psychiatry Clin. Neurosci..

[53] Sibai A.M., Chaaya M., Tohme R.A., Mahfoud Z., Al-Amin H. (2008). Validation of the Arabic version of the 5-item WHO well being index in elderly population. Int. J. Geriatr. Psychiatry.

[54] Barua A., Kar N. (2010). Screening for depression in elderly Indian population. Indian J. Psychiatry.

[55] Awata S., Bech P., Koizumi Y., Seki T., Kuriyama S., Hozawa A., Ohmori K., Nakaya N., Matsuoka H., Tsuji I. (2006). Validity and utility of the Japanese version of the WHO-Five Well-Being Index in the context of detecting suicidal ideation in elderly community residents. Int. Psychogeriatr..

[56] Heun R., Burkart M., Maier W., Bech P. (1999). Internal and external validity of the WHO Well-Being Scale in the elderly general population. Acta Psychiatr. Scand..

[57] Calvente M.D.M.G., Rodrigo M.L.J., Morante E.M. (2010). Guía Para Incorporar la Perspectiva de Género a la Investigación en Salud.

[58] Pardo A., Ruiz M.A. (2002). SPSS 11. Guía Para el Análisis de Datos.

[59] Lennartsson C., Silverstein M. (2001). Does Engagement With Life Enhance Survival of Elderly People in Sweden? The Role of Social and Leisure Activities. J. Gerontol. Ser. B.

[60] Warr P., Butcher V., Robertson I. (2004). Activity and psychological well-being in older people. Aging Ment. Health.

[61] Satorres E. (2013). Bienestar Psicológico en la Vejez y su Relación con la Capacidad Funcional y la Satisfacción Vital. Ph.D. Thesis.

[62] Triadó M.C., Villar P.F. (2003). Envejecer en Entornos Rurales.

[63] Veenhoven R. (1994). El estudio de la satisfacción con la vida. Interv. Psicosoc..

[64] Diener E. (1994). El bienestar subjetivo. Psychosoc. Interv..

[65] Diener E., Suh E.M., Lucas R.E., Smith H.L. (1999). Subjective well-being: Three decades of progress. Psychol. Bull..

[66] Barrientos J. (2005). Calidad de Vida: Bienestar Subjetivo.

[67] Van Hoof J., Kazak J.K. (2018). Urban ageing. Indoor Built Environ..

[68] Mansfield P.K., Preston D.B., Crawford C.O. (1988). Rural-urban differences in women’s psychological well-being. Health Care Women Int..

[69] Bosch P.M., Gómez A.D.V., Ferrer B.S. (2009). Los Grandes Olvidados: Las personas mayores en el entorno rural. Psychosoc. Interv..

[70] Bosch P.M., Gómez A.D.V. (2010). Las personas mayores como actores en la comunidad rural: Innovación y empowerment. Athenea Digit..

[71] Yasuda N., Zimmerman S.I., Hawkes W., Fredman L., Hebel J.R., Magaziner J. (1997). Relation of Social Network Characteristics to 5-Year Mortality among Young-Old versus Old-Old White Women in an Urban Community. Am. J. Epidemiol..

[72] Bolan M. (1997). The Mobility Experience and Neighborhood Attachment. Demography.

[73] Ortiz L.P. (2006). Jubilación, Género y Envejecimiento. Envejecimiento Activo, Envejecimiento en Positivo.

[74] Abellán A., Puyol R. (2006). Envejecimiento y Dependencia: Una Mirada al Panorama Futuro de la Población Española.

[75] Lu L., Shih J.B., Lin Y.Y., Ju L.S. (1997). Personal and environmental correlates of happiness. Pers. Individ. Differ..

[76] Young A.F., Russell A., Powers J.R. (2004). The sense of belonging to a neighbourhood: Can it be measured and is it related to health and well being in older women?. Soc. Sci. Med..

[77] Ponce M.S.H., Rosas R.P.E., Lorca M.B.F. (2014). Social Capital, Social Participation and Life Satisfaction among Chilean Older Adults.

[78] Román X.A.S., Toffoletto M.C., Sepúlveda J.C.O., Salfate S.V., Grandón K.L.R. (2017). Factors associated to subjective wellbeing in older adults. Texto Contexto Enferm..

[79] Pérez-Ortiz L. (2003). Construcción social de la vejez: El sexo y la dependencia. Rev. Española Geriatría Gerontol..

[80] Fericgla J.M. (1992). Envejecer: Una Antropología de la Ancianidad.

[81] Gómez V., De Posada C.V., Barrera F., Cruz J.E. (2007). Factores predictores de bienestar subjetivo en una muestra colombiana. Rev. Latinoam. Psicol..

[82] Lawton M.P., Moss M.S., Winter L., Hoffman C. (2002). Motivation in later life: Personal projects and well-being. Psychol. Aging.

[83] Poon C.Y.M., Fung H.H. (2008). Physical activity and psychological well-being among Hong Kong Chinese older adults: Exploring the moderating role of self-construal. Int. J. Aging Hum. Dev..

[84] García-Molina V.A., Carbonell-Baeza A., Fernández M.D. (2010). Beneficios de la actividad física en personas mayores. Rev. Int. Med. Cienc. Act. Fís. Deporte.

[85] Campos J., Huertas F., Colado J.C., López A.L., Pablos A., Pablos C. (2003). Efectos de un programa de ejercicio físico sobre el bienestar psicológico de mujeres mayores de 55 años. Rev. Psicol. Deporte.

[86] Menec V.H. (2003). The Relation Between Everyday Activities and Successful Aging: A 6-Year Longitudinal Study. J. Gerontol. Ser. B.

[87] Havighurst R.J., Albrecht R. (1954). Older People. Population.

[88] Liechty T., Ribeiro N.F., Yarnal C.M. (2009). Traveled Alone, but Never Felt Alone: An Exploration of the Benefits of an Older Women’s Group Tour Experience. Tour. Rev. Int..

[89] Small J. (2003). The Voices of Older Women Tourists. Tour. Recreat. Res..

[90] Morgan N., Pritchard A., Sedgley D. (2015). Social tourism and well-being in later life. Ann. Tour. Res..

[91] Norstrand J.A., Glicksman A., Lubben J., Kleban M. (2012). The Role of the Social Environment on Physical and Mental Health of Older Adults. J. Hous. Elder..

[92] Vega M.T., Pereira M.A. (2012). Sentido de comunidad y bienestar en usuarios de asociaciones sociales de salud. Glob. J. Community Psychol. Pract..

[93] Phillipson C., Bernard M., Phillips J., Ogg J. (1999). Older people’s experiences of community life: Patterns of neighbouring in three urban areas. Sociol. Rev..

[94] McMUNN A., Nazroo J., Wahrendorf M., Breeze E., Zaninotto P. (2009). Participation in socially-productive activities, reciprocity and wellbeing in later life: Baseline results in England. Ageing Soc..

[95] Hess A. (2018). Gastronomic Societies in the Basque Country. Learning Organizations.

[96] Teixidor F.L. (2018). Los marcos de la sociabilidad en el país Vasco contemporáneo. Vasconia.

[97] Nieboer A.P., Lindenberg S., Boomsma A., Bruggen A.C.V. (2005). Dimensions Of Well-Being And Their Measurement: The Spf-Il Scale. Soc. Indic. Res..

